# New Publications

**Published:** 1898-01

**Authors:** 


### NEW PUBLICATIONS.

A TEXT-BOOK ON HORSESHOEING. By A. LUNGWITZ, Director of the Shoeing School of the Royal Veterinary College, Dresden, Germany. Translated from the eighth German edition by JOHN W. ADAMS, A.B.V., M.D., Professor of Surgery and Lecturer on Shoeing in the Veterinary Department of the University of Pennsylvania. Philadelphia: J. B. Lippincott Company.

A WORK that has successfully passed through so many editions in its original language leaves but little to say for it that has not been oft repeated of its merits and worth. Its greatest need was that of being placed at the command of the English-speaking world, and this has been done in an excellent manner by Prof. Adams, and brought out in attractive shape by the Messrs. Lippincott, whose touch to a new book always gives it value and adds much to its pleasing appearance.

In addition it may be equally well said that what it contains is of the most needful kind, and from student to veterinarian, from apprentice to master, it will be of more service than any book of its kind yet published in the English language. Of the several books already published on horseshoeing in our country, it may be said of them as a whole, that they are based upon one man's personal ideas and deductions, and are not safe or valuable as books of instruction.

From the objects of shoeing, the anatomy of the foot, its relation to the entire limb, the shoeing of healthy feet, hoof care, the foot lesions that require pathological shoeing, it is replete with valuable fundamental knowledge and instruction that must be a great factor in the better education of the shoeing craft in the future, and wipe away many false notions and prejudices that have been handed down in this occupation for many generations. We are sure there await it a wide sale and a generous appreciation at the hands of a body of craftsmen who are advancing this occupation to a much higher and better standing in this country than it has ever occupied before.

---

VETERINARY OPHTHALMOLOGY. By GEORGE G. VAN MATER, M.D., D.V.S. Published by W. R. Jenkins, of New York.

THIS contribution to veterinary literature will, we trust, lead to one more comprehensive and better fitted to meet the requirements of the everyday practitioner. While it has many valuable points, it has followed too closely the lines of human pathology, anatomy, and physiology. The many important diseases of the equine and canine species afford a field of much study and importance, and we trust that this book may be followed by one more extensive and more closely allied to comparative pathology of the lesions of this important member. The book is excellently printed, freely illustrated, and issued in very acceptable form by this well-known publishing-house.

---

*The Philadelphia Medical Journal* made its initial bow to the medical world on January 1st. Its typographical appearance is pleasing to the eye Its well-filled pages of interesting matter from some of the ablest exponents of clinical medicine, with a very well-prepared digest of the contents of the leading medical and surgical journals at home and abroad, matters of general interest carefully collected, promise for its readers a periodical of high merit and usefulness. Its aims and purposes, tersely but clearly stated in its editorial columns, are for a higher and purer practice of legitimate medicine, and "to aid in certain greatly needed reforms, among others to organize and systematize the great coming war against infectious diseases, and especially against tuberculosis, which must be stamped out, utterly eradicated from civilized communities. Veterinary medicine has become a noble and beneficial calling. A national department of public health." These aims and purposes will insure it a support in every way worthy of the projectors of this new journal, many of whom are counted among the foremost in every reform, scientific, educational, professional, political and commercial in our city, State and nation. There is room for it.

---

The *Veterinary Blue-Book*, now announced for publication on January 10th, will prove a very valuable contribution to the busy veterinarian. The large amount of everyday information it will contain will make it valuable for consultation and ready reference, that insures it a welcome upon the desk of every progressive member of the profession.

